# Geriatric Proximal Femur Fractures During the Covid-19 Pandemic - Fewer Cases, But More Comorbidities

**DOI:** 10.1177/21514593211009657

**Published:** 2021-04-29

**Authors:** Christina Polan, Heinz-Lothar Meyer, Manuel Burggraf, Monika Herten, Paula Beck, Henrik Braitsch, Lars Becker, Carsten Vogel, Marcel Dudda, Max Daniel Kauther

**Affiliations:** 1 Department of Trauma, Hand and Reconstructive Surgery, 39081 University Hospital Essen, Essen, Germany; 2 Central Department of Medical Controlling, 39081 University Hospital Essen, Essen, Germany

**Keywords:** geriatric medicine, corona virus, geriatric trauma, fragility fractures, aging, osteoporosis, trauma surgery, emergency, frailty, orthopedics

## Abstract

**Background:**

The COVID-19 pandemic is challenging healthcare systems worldwide. This study examines geriatric patients with proximal femur fractures during the COVID-19 pandemic, shifts in secondary disease profile, the impact of the pandemic on hospitalization and further treatment.

**Methods:**

In a retrospective monocentric study, geriatric proximal femur fractures treated in the first six months of 2020 were analyzed and compared with the same period of 2019. Pre-traumatic status (living in a care home, under supervision of a legal guardian), type of trauma, accident mechanism, geriatric risk factors, associated comorbidities, time between hospitalization and surgery, inpatient time and post-operative further treatment of 2 groups of patients, aged 65-80 years (Group 1) and 80+ years (Group 2) were investigated.

**Results:**

The total number of patients decreased (70 in 2019 vs. 58 in 2020), mostly in Group 1 (25 vs. 16) while the numbers in Group 2 remained almost constant (45 vs. 42). The percentage of patients with pre-existing neurological conditions rose in 2020. This corresponded to an increase in patients under legal supervision (29.3%) and receiving pre-traumatic care in a nursing home (14.7%). Fractures were mostly caused by minor trauma in a home environment. In 2020, total number of inpatient days for Group 2 was lower compared to Group 1 (p = 0.008). Further care differed between the years: fewer Group 1 patients were discharged to geriatric therapy (69.6% vs. 25.0%), whereas in Group 2 the number of patients discharged to a nursing home increased.

**Conclusions:**

Falling by elderly patients is correlated to geriatric comorbidities, consequently there was no change in the case numbers in this age group. Strategic measures to avoid COVID-19 infection in hospital setting could include reducing the length of hospital stays by transferring elderly patients to a nursing home as soon as possible and discharging independent, mobile patients to return home.

## Introduction

In 2020, a viral disease now known as COVID-19, caused by the coronavirus SARS-CoV-2, developed into a worldwide pandemic.^1-3^ Elderly patients, especially those with comorbidities, are one of the groups with the highest risk of becoming infected and dying from the disease.^4,5^ The loss of bone mass in elderly people due to osteoporosis increases the likelihood of fractures which can occur as the result of even minor trauma or tripping over.^6^ An especially high mortality rate has been observed among geriatric patients with a combination of COVID-19 disease and a fracture of the proximal femur.^7,8^

In the geriatric population the tendency to fall is due to various intrinsic and extrinsic risk factors. The process of aging leads to frailty syndrome, a multifactorial decline in health and function including decreasing intelligence/imagination, orthostatic dysfunction, impaired visual performance and balance, weakness and instability of the musculoskeletal system and reduced mobility, which may also be due to anxiety.^9,10^ Furthermore, limited perception in the context of mild cognitive impairment (MCI), dementia or delirium as well as diseases such as Parkinson’s disease, syncope and many more can also increase the probability of falling.^9,11,12^ Polypharmacy is another factor often found in elderly people. Potential side-effects of medication or interaction between different medicines can cause to a change in gait behaviour, leading to falls.^11^ While anticoagulation therapy is required for many concomitant geriatric diseases, after a fall it can trigger severe bleeding complications.^12^

A marked drop in the overall number of orthopedic outpatients and inpatients was seen during the first phase of the corona pandemic.^13,14^ Especially among young people, the number of injuries to the upper extremity due to work, sports and traffic accidents decreased due to a reduction in the volume of traffic and suspension of team sports, while injuries caused by accidents in the home increased.^15^

In this study, we investigated the case numbers of geriatric patients with proximal femur fractures during the first wave of the COVID-19 pandemic compared to 2019, shifts in comorbidities and the impact of the pandemic on hospitalization and further treatment.

## Methods

### Study Design

This monocentric retrospective data analysis included the case numbers of geriatric proximal femoral fractures for the first half (H1) of 2019 and 2020, i.e. femoral neck fractures, pertrochanteric fractures and subtrochanteric fractures. The patients were divided into 2 groups according to age: between 65 and 80 years (Group 1) and over 80 years of age (Group 2). Exclusion criteria were age under 65 years, fractures of the femoral shaft or distal femur as well as periprosthetic fractures and septic conditions. To the best of our knowledge, no patients were infected with COVID-19 during the investigated time periods.

### Ethics

The study was approved by the Ethics Committee of the Medical Faculty of the University of Duisburg-Essen, Germany (approval number 20-9457-BO). In addition, this study was conducted in accordance with the principles of the World Medical Association as laid down in the Declaration of Helsinki and the national regulations for the conduct of clinical studies.

### Assessment of Study Parameters

The following patient parameters were collected: gender, month of accident, age, geriatric risk factors including history of cardiological, nephrological, oncological and neurological concomitant diseases. Cardiological disorders included heart attack, coronary heart disease, cardiac arrhythmia as well as heart failure. Neurological disorders included Parkinson’s disease, stroke, epilepsy, intracranial bleeding and others. Known risk factors for falling such as polypharmacy with more than five documented drugs, anticoagulation or the presence of a neurological or cardiac disease were assessed. Pre-traumatic care at home or in a nursing home and the presence of a legal guardian were evaluated as indications of an already existing reduction in autonomy. The trauma mechanism, the type of trauma and the location of the accident were determined (domestic environments / on the street or in a hospital). Additionally, the days between admission and operative treatment, the total time of inpatient care, time in the intensive care and normal wards and the total length of stay in hospital were considered. Furthermore, the place of postoperative follow-up treatment was recorded: either in the patient’s own home, a nursing home, geriatric specialty care or another department. Finally, the number of patients who died in hospital was analyzed.

### Statistical Analysis

For the statistical analysis, the SPSS 27 software was used (IBM, New York, NY, USA). Descriptive statistics revealed the mean, standard deviation, median, interquartile range and the 2-sided 95% confidence interval. The calculations included the percentage increase or decrease of the mean values over the time periods. After testing for normal distribution, the non-parametric values were analyzed using Mann-Whitney U tests of independent samples. A p-value <0.05 was defined as statistically significant.

## Results

### Incidence Shift of Femur Fractures

**Figure 1.**
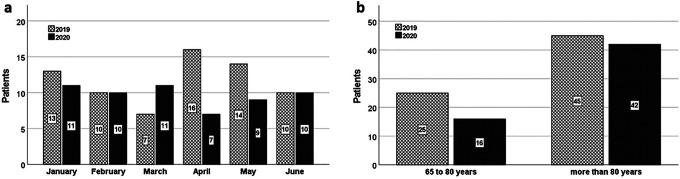
a) Patient numbers in the first six months (January - June) of 2019 (H1 2019) and 2020 (H1 2020). b) Age distribution of the patients in Group 1 (65 – 80 years) and Group 2 (over 80 years).

Comparison of the first half of the years 2019 and 2020 revealed a distinct difference in the number of cases of geriatric proximal femur fractures during the lockdown in April and May 2020 compared to the previous year (Figure 1a). A total of 70 patients older than 65 with proximal femur fractures were treated in the first half of 2019, compared to only 58 in 2020. The number of patients in Group 1 declined from 25 in 2019 to 16 in 2020, while the number of very elderly patients in Group 2 remained almost constant (45 vs. 42) (Figure 1b).

### Pre-Traumatic Comorbidities and Pre-Clinical Factors

In H1 2020, there was a rise in the number of geriatric patients with proximal femur fractures who also had a secondary disease profile compared to H1 2019 (Table 1).

**Table 1.** Patients’ Pre-Traumatic Status in Terms of Comorbidities and Pre-Clinical Situation.^a^

Year		2019				2020				% Change 2019 to 2020
Group		Total	*[%]*	65-80 yrs	80+ yrs	Total	*[%]*	65-80 yrs	80+ yrs	
Patient number		n = 70		n = 25	n = 45	n = 58		n = 16	n = 42	*[%]*
Comorbidities & medication	Neurological disorders	19	*27.1*	9	10	30	*51.7*	7	23	*90.6*
	Dementia	17	*24.3*	3	14	17	*29.3*	3	14	*20.7*
	Vertigo	2	*2.9*	1	1	4	*6.9*	0	4	*141.4*
	Heart diseases	40	*57.1*	11	29	34	*58.6*	5	29	*2.6*
	Hypertension	56	*80.0*	21	35	52	*89.7*	15	37	*12.1*
	Atrial fibrillation	22	*31.4*	6	16	15	*25.9*	2	13	*-17.7*
	Diabetes mellitus	14	*20.0*	5	9	9	*15.5*	6	3	*-22.4*
	Renal Failure	14	*20.0*	5	9	9	*15.5*	3	6	*-22.4*
	Osteoporosis	10	*14.3*	2	8	6	*10.3*	0	6	*-27.6*
	Tumor disease	11	*15.7*	5	6	15	*25.9*	4	11	*64.6*
	Polypharmacy	47	*67.1*	17	30	47	*81.0*	11	36	*20.7*
	Anticoagulants	42	*60.0*	14	28	38	*65.5*	12	26	*9.2*
Pre-clinical situation	Legal supervision	14	*20.0*	2	12	15	*25.9*	4	11	*29.3*
	Care in nursing home	20	*28.6*	3	17	19	*32.8*	3	16	*14.7*
	Private home	50	*71.4*	22	28	39	*67.2*	13	26	*-5.8*
	Fall in home situation	57	*81.4*	17	40	47	*81.0*	11	36	*-0.5*
	Minor trauma	67	*95.7*	23	44	55	*94.8*	14	41	*-0.9*

^a^ Number of Patients, Percentage [%] and Percentage Change between the Years 2019 to 2020. The Percentage Change Equals the Change in Value Divided by the Absolute Value of the Original Value Minus 1 and Multiplied by 100. Positive Numbers Represent a Percentage [%] Increase and Negative Numbers a Percentage [%] Decrease.

The relative proportion of patients with neurological secondary diagnoses almost doubled, increasing from 27.1% in 2019 to 51.7% in 2020. In 2019, 24.3% of patients with proximal femur fracture suffered from dementia compared to 29.3% in 2020. The relative proportion of patients with a history of vertigo also increased from 2.9% in the first half of 2019 (H1 2019) to 6.9% in the first half of 2020 (H1 2020). The percentage of patients with pre-existing neurological conditions (neurological disorders (90.6%), dementia (20.7%) and vertigo (141.4%)) increased in the pandemic situation, which corresponded to more patients under legal supervision (29.3%) and receiving pre-traumatic care in a nursing home (14.7%). In contrast, in both age groups there were fewer patients, who did not have neurological diseases.

The vast majority of geriatric patients with proximal femur fractures suffered from arterial hypertension, with 80.0% in H1 2019 and 89.7% in H1 2020. As far as heart diseases were concerned, the relative proportion of patients with congestive heart failure, valvular heart failure and heart attacks remained almost constant with 57.1% in 2019 and 58.6% in 2020. However, the proportion of patients with atrial fibrillation decreased slightly from 31% in 2019 to 26% in 2020. Additionally, the relative proportion of patients with diabetes mellitus, renal failure and osteoporosis declined slightly in 2020. Patients with a medical history of cancer presented in 25.9% of the cases in 2020 compared to 15.7% in 2019, which indicated a percentage rise of 64.6%. The rise in the percentage of patients with polypharmacy (treatment with five or more prescribed drugs) was 20.7%, affecting 67.1% of the patients in 2019 and 81.0% in 2020. A slight increase was found regarding anticoagulation, which was taken by 60.0% of all patients with proximal femur fractures in 2019 and by 65.5% in 2020 (Table 1).

In both years, the vast majority (about 95%) of proximal femur fractures occurred as result of minor trauma and mostly (about 80%) in the home environment (nursing care or private) (Table 1). The proportion of patients under legal supervision increased from 20.0% in H1 2019 to 25.9% in H1 2020. Equally, the proportion of patients coming from nursing care homes increased from 28.6% in H1 2019 to 32.8% in H1 2020, a percentage rise of 14.7% (Table 1).

### Inpatient Time

**Figure 2.**
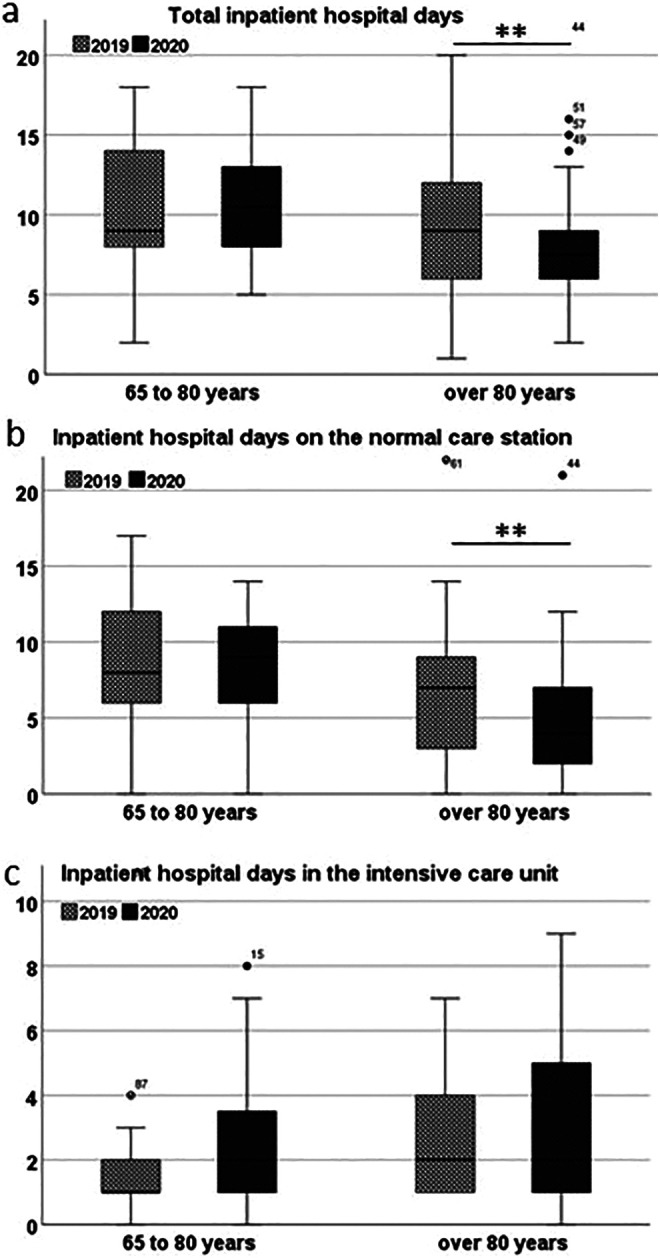
Comparison of the hospital stays in days in the first half of 2019 with the first half of 2020 (the first wave of the Covid-19 pandemic). a) In total, b) in the normal care unit, c) in the intensive care unit (ICU). The box plots span the interquartile range. The vertical line inside the box represents the mean. The whiskers extend to the highest and lowest observations.

The average total length of stay in hospital of both groups of geriatric patients with proximal femur fracture did not change in H1 2020 compared to H1 2019. However, in 2020 the total length of hospital stay of patients in Group 2 was shorter than that of Group 1 patients (p=0.008) (Table 2) (Figure 2). Comparison of the inpatient days on the normal ward showed that the very elderly patients of Group 2 stayed in a normal ward for a significantly shorter time compared to the younger patients of Group 1 in H1 2020 (p=0.001). In contrast, there was no significant difference in the length of stay in the ICU.

**Table 2.** Number of Inpatient Hospital Days in Total, in a Normal Care Unit and in an ICU as Well as the Time Between Admission and Surgery.^a^

	2019	2020		2019			2020		
Year Groups	Both groups		p-value	65-80 yrs	80+ yrs	p-value	65-80 yrs	80+ yrs	p-value
Inpatient hospital days (total)	9.9 ± 5.3	9.0 ± 4.4	0.213	10.4 ± 4.2	9.7 ± 5.8	0.350	10.9 ± 3.8	8.3 ± 4.4	0.008
	(8.7, 11.2)	(7.9, 10.2)		(8.6, 12.1)	(8.0, 11.4)		(8.9, 13.0)	(6.9, 9.7)	
Inpatient hospital days (normal care unit)	7.1 ± 4.8	5.8 ± 4.3	0.088	8.2 ± 4.6	6.5 ± 4.8	0.163	8.4 ± 3.7	4.8 ± 4.1	0.001
	(6.0, 8.3)	(4.7, 6.9)		(6.3, 10.1)	(5.1, 8.0)		(6.4, 10.4)	(3.5, 6.1)	
Inpatient hospital days (ICU)	2.8 ± 4.1	3.2 ± 3.4	0.129	2.2 ± 3.3	3.2 ± 4.5	0.057	2.6 ± 2.2	3.5 ± 3.7	0.550
	(1.8, 3.8)	(2.3, 4.1)		(0.9, 3.6)	(1.8, 4.5)		(1.4, 3.8)	(2.3, 4.6)	
Days between admission and surgery	0.9 ± 0.9	0.8 ± 1.1	0.291	1.0 ± 0.7	0.8 ± 1.0	0.185	1.3 ± 1.8	0.6 ± 0.6	0.405
	(0.7, 1.1)	(0.5, 1.1)		(0.7, 1.2)	(0.5, 1.1)		(0.4, 2.3)	(0.5, 0.8)	

^a^The data present the mean ± standard deviation as well as the 95% confidence interval.

### Post-Hospital Care

**Figure 3.**
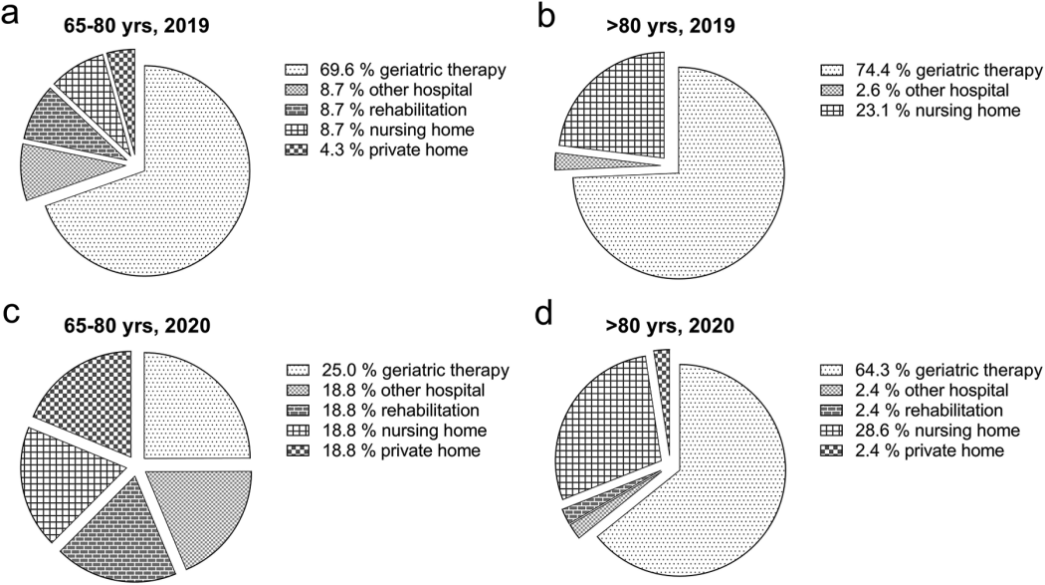
Post-inpatient status: a) n=23 and b) n=39 in H1 2019, and c) n= 16 and d) n=42 in H1 2020.

Further care after discharge differed between the years: in H1 2019 most of the patients in Group 1 went to geriatric speciality care (69.6%) compared to only 25.0% in H1 2020. The proportion of patients in Group 2 whose post-hospital care was performed in a nursing home increased (23.1% vs. 28.6%), while geriatric specialty care in this group decreased from 74.4% in 2019 to 64.3% in 2020 (Figure 3).

There were 2 deaths in Group 1 and six in Group 2 in 2019, but no inpatient deaths following proximal femur fractures in orthopedic trauma surgery were recorded in 2020. However, in 2020 one Group 2 patient was discharged to return home for palliative conservative therapy due to advanced underlying diseases.

## Discussion

In this study, we found a lower number of geriatric patients with proximal femur fractures during H1 2020, which coincided with the first wave of the COVID-19 pandemic in Germany, compared to H1 2019. Regarding the age groups, the number of patients in Group 1 (65-80 years) decreased (25 vs. 16), while the number of very elderly patients in Group 2 (80 + years) remained almost constant (45 vs. 42). In both age groups, there were fewer patients without neurological diseases than in 2019. The proportion of patients with comorbidities like pre-existing neurological conditions increased in the pandemic situation, which was reflected by the larger number of patients under legal supervision and receiving care in a nursing home prior to trauma.

The lower numbers could be partially due to the decline in road traffic and traffic-associated accidents during the first COVID lockdown.^16^ However, most of the geriatric proximal femur fractures included in this study occurred as the result of minor trauma (95%) and in the home environment (81%) without the direct influence of road traffic. It was observed that during the onset of the COVID-19 pandemic not only the overall number of trauma patients presenting in the emergency room decreased significantly,^14^ but also that of patients with heart attacks and strokes.^17-20^ Nevertheless, it is highly unlikely that geriatric patients with proximal femur fractures would not present in the emergency department as this injury is usually associated with intolerable pain and immobility.

During the lockdown, the residents in nursing homes in Germany were completely isolated from their families and visitors as they are at an especially high risk of infection and mortality.^21,22^ As a result, there has been much public discussion about the effects of isolation and reduced mobilization due to contact restrictions and physical distancing. However, according to our data, there was no relevant change in the number of very elderly patients. This could indicate that at least some degree of mobilization has been maintained in this age group. Although speculative, we suppose that even elderly patients (65-80 years) reduced their daily activities during the lockdown, leading to fewer fractures in this group. On the other hand, the very frail patients, especially those with neurological comorbidities, remained at risk for proximal femoral fractures.

Overall, a higher relative proportion of pre-morbid patients with neurological diseases such as dementia, stroke and vertigo was observed in H1 2020. As the risk of falling is generally high for elderly patients with geriatric comorbidities the number of falls due to neurological disabilities or syncope is unlikely to be influenced by changes in lifestyle made necessary by lockdown measures and can therefore be expected to remain constant.^9,10,23,24^

In the present study, the total number of inpatient days of the very elderly patients of Group 2 was significantly lower compared to Group 1. This difference was due to earlier transfer to geriatric departments or discharge to a nursing home as a strategic measure to avoid COVID-19 infection of geriatric patients in our hospital setting. The younger patients were more frequently sent home instead of being transferred to local geriatric departments as the capacity of these departments was reduced during the first pandemic wave. Furthermore, the length of stay in the ICU did not differ significantly between the investigated time periods. This indicates on the one hand that ICU capacity is needed for the care of these patients and, on the other hand, that there was no reduction in ICU availability for these patients during the lockdown, at least in our department.

There was a defined standard operation plan for patients with proximal femur fractures and assumed or confirmed COVID-19 infection. This included a screening for COVID-19 symptoms with a questionnaire, fever measurement as well as an emergency room, an operating room and isolation area on the ward in reserve, especially for these patients.

The new measures of the anaesthesia plan were necessary, but did not have to be applied. None of the patients included in the study had a suggested or confirmed COVID-19 infection during this time period. Therefore, there was no increased risk of thromboembolic events due to COVID-19 in the study population. This might explain why we did not find any differences in postoperative complications.

We have investigated the length of stay and could show that the very elderly patients had a shorter in-hospital time during pandemic. They had to be negative for COVID-19 before admission to the nursing homes. We scheduled these tests 2 days before the expected admission so that the results were present in time. Since we did not have any positive cases, we could keep the in-patient times as short as possible. Our study has a number of limitations, first and foremost its finite time interval and monocentric character. The differences in comorbidities is highly likely to be correlated with the reduced activity of the patients and their fear of being treated in hospital with the risk to infections but could also be coincidence. Furthermore, changes in the risk behaviour of the population and different strategic approaches by hospitals and nursing homes may influence geriatric case numbers. Studies should be performed to investigate the potential consequences of changes in discharge strategy under these specific conditions.

## Conclusion

For elderly patients the risk of falling is increased by geriatric comorbidities, especially neurological disorders, dementia and vertigo. Therefore, a significant change in the numbers of these patients is not to be expected during a lockdown. However, adequate ICU resources for their treatment must be provided as usual.

Like the population in general, patients with fewer or no comorbidities have obviously reduced their mobility during the lockdown, which has led to a decrease in accidents and falls.

Potential measures to deal with reduced hospital resources during the COVID-19 pandemic could include the concept of keeping inpatient stays as short as possible. In our hospital, we transferred the very elderly patients to geriatric specialty care as far as capacity was available, whereas patients in the younger age group mostly returned to their own home after discharge.
